# Influence of Cold Stress on Physiological and Phytochemical Characteristics and Secondary Metabolite Accumulation in Microclones of *Juglans regia* L.

**DOI:** 10.3390/ijms25094991

**Published:** 2024-05-03

**Authors:** Nina V. Terletskaya, Elvira A. Shadenova, Yuliya A. Litvinenko, Kazhybek Ashimuly, Malika Erbay, Aigerim Mamirova, Irada Nazarova, Nataliya D. Meduntseva, Nataliya O. Kudrina, Nazym K. Korbozova, Erika D. Djangalina

**Affiliations:** 1 Faculty of Biology and Biotechnology, Al-Farabi Kazakh National University, Al-Farabi 71, Almaty 050040, Kazakhstan; malika.isa99@mail.ru (M.E.); aigerim.mamirova@mail.com (A.M.); kudrina_nat@mail.ru (N.O.K.); naz-ik@mail.ru (N.K.K.); 2 Institute of Genetic and Physiology, Al-Farabi 93, Almaty 050040, Kazakhstan; shadel08@mail.ru (E.A.S.); rumex1978@gmail.com (Y.A.L.); kajeke@mail.ru (K.A.); nat.mdnt@gmail.com (N.D.M.); 3 Faculty of Chemistry, Al-Farabi Kazakh National University, Al-Farabi 71, Almaty 050040, Kazakhstan

**Keywords:** *Juglans regia*, microclones, cold stress, anatomy, metabolome

## Abstract

The current study investigated the impact of cold stress on the morphological, physiological, and phytochemical properties of *Juglans regia* L. (*J. regia*) using in vitro microclone cultures. The study revealed significant stress-induced changes in the production of secondary antioxidant metabolites. According to gas chromatography–mass spectrometry (GC–MS) analyses, the stress conditions profoundly altered the metabolism of *J. regia* microclones. Although the overall spectrum of metabolites was reduced, the production of key secondary antioxidant metabolites significantly increased. Notably, there was a sevenfold (7×) increase in juglone concentration. These findings are crucial for advancing walnut metabolomics and enhancing our understanding of plant responses to abiotic stress factors. Additionally, study results aid in identifying the role of individual metabolites in these processes, which is essential for developing strategies to improve plant resilience and tolerance to adverse conditions.

## 1. Introduction

During the growth season, plants continuously face a wide range of stress factors. Growth, development, and photosynthesis are key physiological indicators of a plant’s ability to thrive and reproduce under adverse conditions [1]. Metabolomics, which studies metabolites in biological systems, provides tools for understanding and valuable insights into plant stress responses and the role of individual metabolites [2,3,4,5,6]. Unlike the transcriptome and proteome, the metabolome is not always directly connected to the plant genome [7]. The systematic identification and quantification of metabolites reveal the organism’s biochemical reactions to specific conditions and help define the phytochemical profile of the phenotype [8,9,10].

Gas chromatography–mass spectrometry (GC–MS) is a powerful analytical tool in metabolomics for identifying and quantifying both volatile and non-volatile compounds, including bioactive substances, in plant samples. This technique provides critical insights into the physiological state of plants [5]. Due to its high sensitivity, GC–MS can detect minute amounts of metabolites within complex plant matrices, enabling researchers to explore the dynamics of metabolic changes during various growth stages and in response to environmental stimuli, with excellent resolution and reproducibility. The application of GC–MS to profile stress-responsive metabolites, which help plants adapt to adverse conditions, is crucial in contemporary biotechnology research [11,12,13].

Stress factors affect different species, and even individual plants within the same species at different developmental stages, in various ways. In some cases, these effects can be beneficial, eliciting desired responses in the crops under study [14,15]. Indeed, the production of beneficial secondary metabolites in medicinal plants can be enhanced [16,17,18,19,20,21]. Today, there is a growing global interest in exploring how plants respond at the metabolomic level to cold stress [22,23,24,25,26,27]. Research indicates that low-temperature stress can effectively stimulate the production and accumulation of secondary metabolites, both in vivo and in vitro [28,29,30]. However, the specific mechanisms by which cold stress stimulates secondary metabolism in medicinal plants remain unclear and warrant further investigation.

The walnut (*Juglans regia* L.) is among the medicinal plants of interest [31,32,33] due to its rich content of antioxidants such as flavonoids, phenolic acids (notably ellagic acid), melatonin, folate, gamma-tocopherol (vitamin E), selenium, juglone, and proanthocyanidins [34,35,36,37] in various organs (kernels, shells, roots, and leaves). Several studies have shown that *J. regia*, along with its extracts and essential oils, exhibits a range of biological and pharmacological properties, including antibacterial, antifungal, antioxidant, and anti-inflammatory activities, demonstrating effectiveness against a broad spectrum of pathogens [38,39,40,41,42]. Innovative modern biotechnologies are increasingly focused on developing medicinal crops that are rich in biologically active compounds via manipulating growth conditions to enhance the production of these valuable compounds.

The microclonal propagation of walnuts has advanced significantly in recent years. Researchers have primarily focused on identifying the optimal mineral and hormonal compositions of nutrient media, selecting the appropriate type of explants, and establishing cultivation conditions. These efforts aim to develop effective protocols for in vitro walnut propagation at the commercial scale [43,44,45,46,47,48]. Despite these advancements, studies on the chemical composition of *J. regia* micro-shoots remain limited. However, the presence of naphthoquinones such as hydro-juglone glucoside and juglone, as well as flavonoids like myricitrin and quercitrin, has been confirmed in walnut micro-shoots, and inter-varietal differences in these compounds have been identified [49,50]. Consequently, walnut microclones represent a promising source of biologically active chemicals and provide a valuable model for studying various morphophysiological and phytochemical processes in plant tissues.

The current study aimed to examine the impact of cold stress on the anatomical–morphological, photosynthetic, and phytochemical characteristics of *J. regia* microclones. There is a lack of research on how the walnut might respond to this type of abiotic stress and the potential benefits of such studies, making this a significant topic for discussion. Consequently, this research explored the physiological and phytochemical properties of *J. regia* shoot microclones in vitro after exposure to low positive temperatures.

Our study delves into how low positive temperatures influence the plasticity of *J. regia* during the initial stages of microclone formation. It offers both theoretical and practical insights into the species’ ecological adaptation strategies, as well as the potential to harness these adaptation processes to develop methods for the targeted synthesis of beneficial secondary metabolites for medical use.

## 2. Results

### 2.1. Microclone Propagation

**Figure 1:**
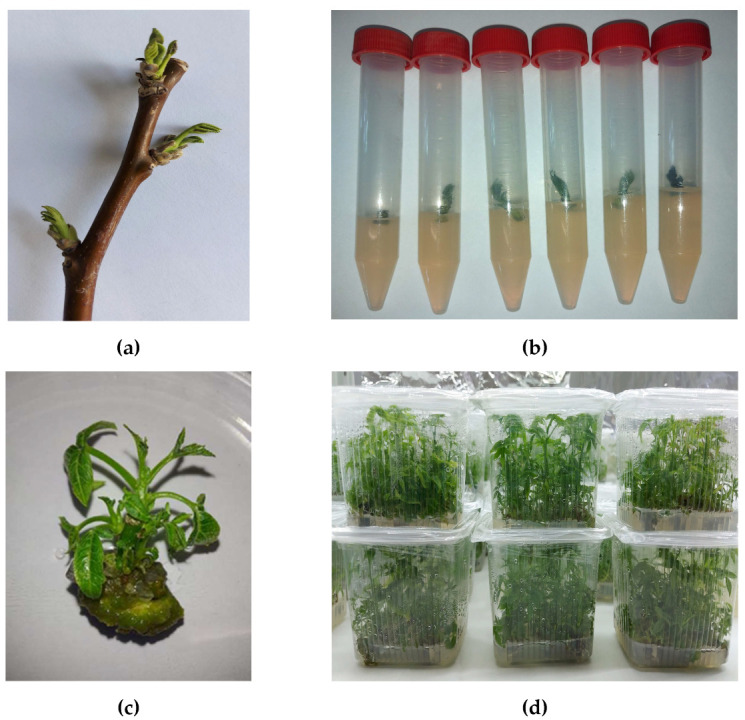
Induction and propagation of *J. regia* shoots: (**a**) lignified branches with dormant buds; (**b**) bud explants on a nutrient medium; (**c**) nodal explant on medium with BAP; (**d**) microclones.

After sterilisation, 82.8 ± 3.6 aseptically viable explants were obtained, while 17.2 ± 1.06 were found to be infected (Figure 1b). Following the application of 6- Benzylaminopurine (BAP) at a concentration of 1 mg L^−1^, small shoots (0.5–0.7 cm) began to form within 7–10 days, accompanied by callus formation at the base of the cut site and the emergence of new buds within the callus, which subsequently developed into additional shoots (Figure 1c). By the 20th day, shoots measuring 0.6–1.2 cm in height had formed, resulting in a reproduction ratio of 1:7 (Figure 1d).

In total, 470 shoots were obtained as a result, 300 shoots of which were then subjected to low positive temperatures, specifically 3–5 °C, referred to as “cold stress”, for 72 h. The remaining 170 shoots were cultivated for mass cloning, some of which will later be transplanted onto a medium to induce root formation. Rooting was carried out on a medium with 0.5 mg L^−1^ indole-3-butyric acid (IBA).

### 2.2. Anatomical Characteristics of Microclones

**Figure 2:**
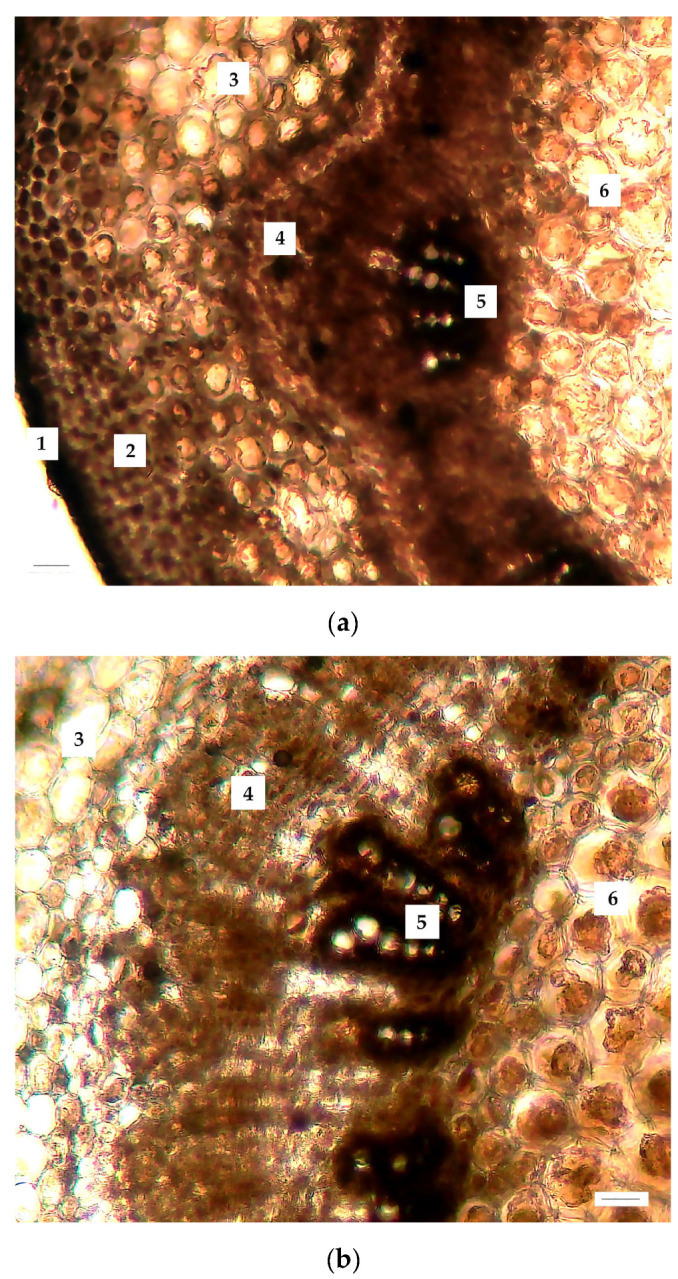
Changes in the anatomical structure of *J. regia* stem exposed to cold stress: (**a**) control and (**b**) cold stress (+3 °C, 72 h). Labels: 1—epidermis; 2—sclerenchyma; 3—parenchyma; 4—phloem; 5—xylem; 6—core. Scale bar = 50 µm.

**Figure 3:**
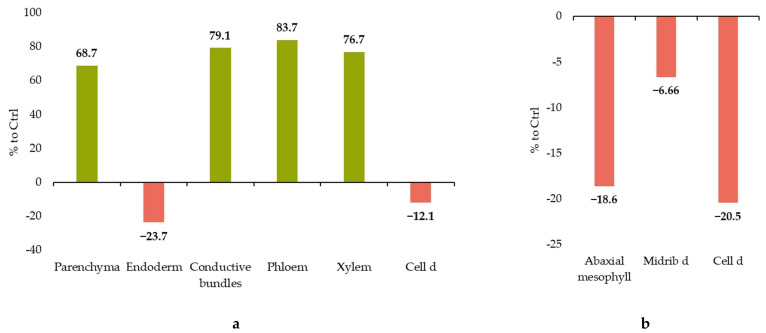
*J. regia* anatomy alterations when exposed to cold stress. (**a**) Stem and (**b**) leaf. Notes: only significant changes are shown.

When the anatomical characteristics of the stems of *J. regia* microclones were examined, considerable changes were seen in the metric parameters of numerous tissues, both in negative and positive dynamics. Thus, considerable increases in the size of vascular bundles were discovered (both phloem (+83% to control) and xylem (+86% to control)), as well as the thickness of parenchyma tissue (+38% to control) (Figure 2 and Figure 3 and Table 1).

**Figure 4:**
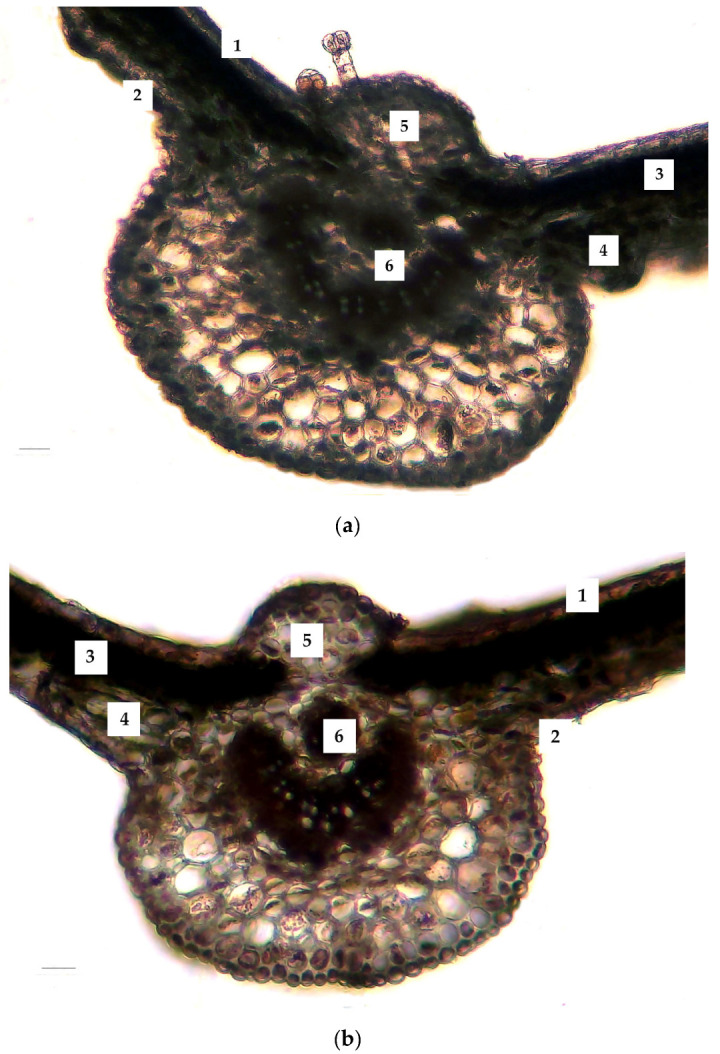
Changes in the anatomical structure of a *J. regia* leaf exposed to cold stress: (**a**) control and (**b**) cold stress (+3–5 °C, 72 h). Labels: 1—adaxial epidermis; 2—abaxial epidermis; 3—palisade mesophyll; 4—spongy mesophyll; 5—central vein; 6—central conductive bundle. Scale bar = 50 µm.

When examining tissue sizes and comparing the characteristics of the leaf’s histological structures, a difference was seen in the indicators of the control and experimental versions, mostly with regard to negative dynamics after cooling (Figure 4 and Table 1). This trend is readily visible in the histogram (Figure 4), which shows that the diameter of parenchyma cells under stress was 85% of the control values, while the thickness of the abaxial epidermis was 84% of the control.

### 2.3. Photosynthetic Characteristics of Microclones

**Figure 5:**
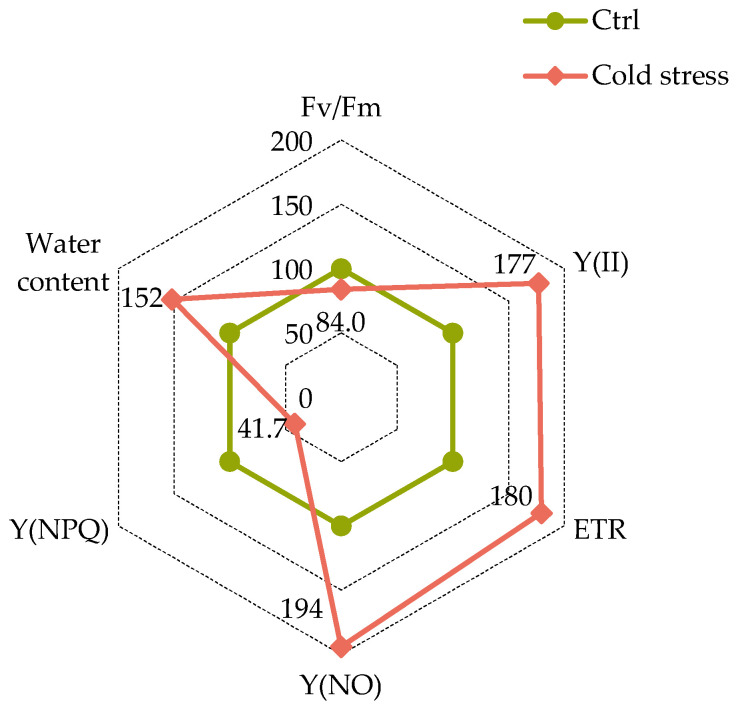
Chlorophyll *a* fluorescence indicator in leaves of *J. regia* exposed to cold stress.

Cold stress had a substantial effect on the photosynthetic characteristics of *J. regia* microclone leaves, as evidenced by a decrease in the amount of photochemical quenching. This determines the maximum quantum efficiency in the dark, Fv/Fm (−13.5% to control), as well as nonphotochemical energy conversion in PSII, due to the downregulation of the light-harvesting function (adjustable dissipation energy) Y(NPQ) (−58.3% to control) under stressful conditions. Meanwhile, there was a significant increase in the electron transport rate (ETR) (+63.1% to control) and the parameter quantum yield of nonphotochemical energy conversion in PSII, other than that caused by the downregulation of the light-harvesting function (unregulated dissipation energy) Y(NO) (+49.7% to control). The water content of plant tissues increased by one and a half times when compared to the control (Figure 5).

### 2.4. Phytochemical Characteristics of Microclones

Thin-layer chromatography (TLC) in various solvent systems with particular developers was used to identify the main groups of bioactive chemicals in extracts from *J. regia* microclones. A comparative study of stress variations revealed that the analysed samples differed in their composition of the major types of secondary metabolites or groups of chemicals. A qualitative analysis of plant metabolites detected in extracts of microclones cultivated under control and stress conditions revealed specific patterns (Table 2).

Flavones, flavanols, anthracene derivatives, and condensed tannins were identified in the analysed extracts. When microclones were treated at low positive temperatures, control values for flavones, phenolic acids, and tannins were found to be higher.

**Figure 6:**
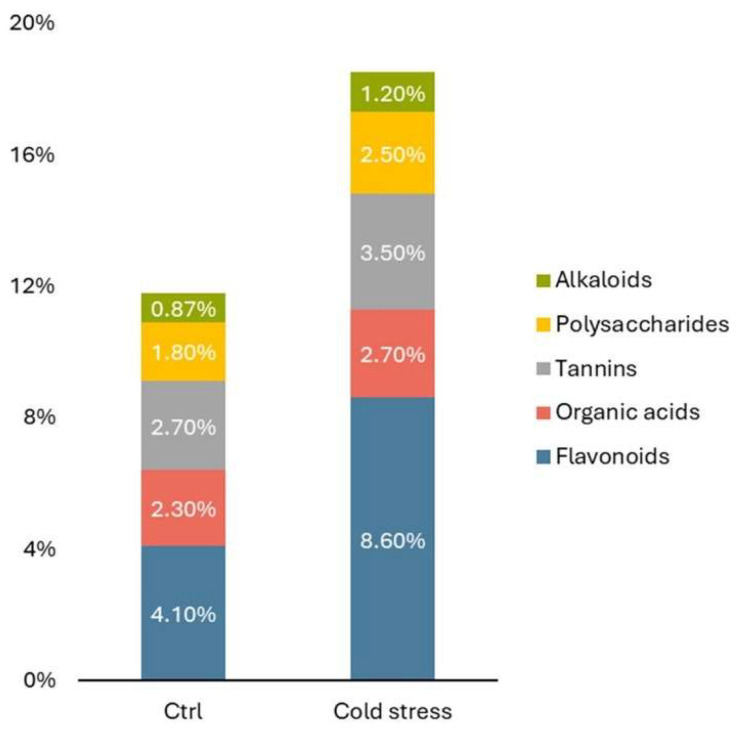
Active compounds content in *J. regia* microclones exposed to cold stress.

The results of the quantitative analysis (Figure 6) confirm the results of the qualitative analysis and suggest that the quantitative content of the main groups of biologically active substances, such as flavonoids, alkaloids, polysaccharides, tannins, and organic acids when treating microclones at low positive temperature increases compared to control samples.

**Figure 7:**
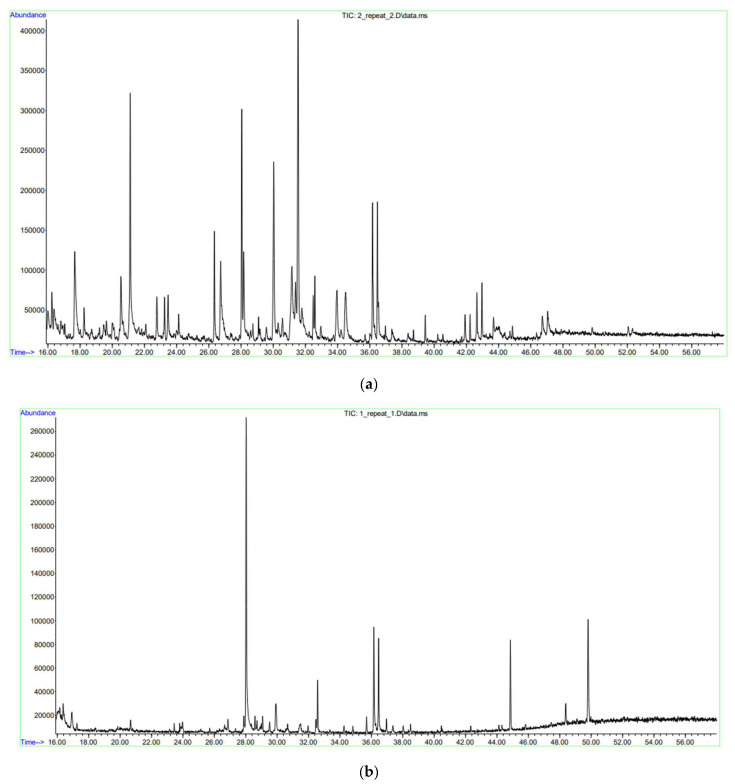
GS–MS chromatogram of *J. regia* microclones’ extract. (**a**) Control and (**b**) cold stress (+3 °C, 72 h).

Based on the results of a detailed analysis via gas chromatography with mass spectrometric detection, 27 compounds were identified in the extract of the leaf stem part of *J. regia* microclones cultivated under control conditions, of which cyclic polyhydric alcohols predominated—1,2,3,5-cyclohexanetetrol (α,2β,3α,5β—cyclohexanetetrol) (15.25%); polyhydric phenols—hydroquinone (9.38%), pyrocatechol (6.40%); naphthoquinones—1,4-naphthalenedione, 5-hydroxy—(7.50%), carbohydrates—ethyl α-d-glucopyranoside (8.29%) and lactones—3-deoxy-d-mannoic lactone (6.98%). The identification of phytochemical compounds was confirmed based on the peak area, retention time and molecular formula (Table 3, Figure 7).

The analysis of the chromatography–mass spectra showed that stress conditions significantly modify the dominant spectrum of PSM of *J. regia* microclones. It was demonstrated that the chromatographic spectra of *J. regia* microclones performed worse than the control under cold stress conditions, although multiple peaks of chemicals were detected that were greatly above the control levels. Thus, according to the results of gas chromatography with mass spectrometric detection, 10 compounds were identified in the nut extract (experiment), of which naphthoquinones predominated—5-hydroxy-1,4-naphthoquinone (juglone) (49.9%); esters of unsaturated fatty acids—ethyl ester of (E)-9-octadecenoic acid (13.7%), ethyl ester of octadecanoic acid (11.1%); ethyl ester of palmitic acid (5.90%); and sulfoxides based on cyclopropanes and benzene—2,3-diphenylcyclopropyl) methylphenyl sulfoxide (10.7%). At the same time, a sevenfold higher content of (2,3-Diphenylcyclopropyl)methyl phenyl sulfoxide and fatty acid esters was noted concerning the control 1,4-Naphthalenedione, 5-hydroxy—(juglone) (Table 3 and Figure 6).

## 3. Discussion

Low temperatures, or cold stress, are a major environmental factor that inhibits the growth and development of many plant species [51,52,53]. Symptoms of stress typically appear 48–72 h after exposure to cold temperatures, although this duration can vary based on the plant type, growth stage, and individual susceptibility to cold. Cold stress induces various phenotypic symptoms such as reduced growth rate, wilting, chlorosis (leaf yellowing), and necrosis (tissue death) in leaves. Additionally, it significantly diminishes plant reproductive capabilities [54]. Cold stress also triggers anatomical and morphological changes compared to the control conditions, impacting physiological and biochemical processes and altering the concentration of biologically active substances in the above-ground organs [55]. These changes in anatomical characteristics are crucial as they underpin the physiological processes within the plant. This is because all adaptive processes are linked to the structures of organelles, cells, and tissues and their spatial relationships within plant tissues [56].

Interestingly, hypothermia does not necessarily reduce biomass accumulation; it can even induce an increase in the water content of plant tissues [57]. During cold stress, the expansion of the stem’s conductive and parenchymal tissues compared to the control is necessary to manage an increased flow of water and organic compounds. This adjustment leads to a higher total water content in the tissues of walnut microclones under cold stress, which may be an indicator of *J. regia* microclones’ adaptation to cold [58,59,60].

Study findings show that different plant organs exhibit distinct responses to cold stress. In the leaf blade tissues, a more “compressed” structure under stress was observed, where the diameter of the parenchyma cells was reduced to 85% of the control values and the thickness of the abaxial epidermis was 84% of the control. Conversely, there was a 15% increase in the thickness of the mesophyll, largely due to the thickening of the spongy tissue layer compared to the control. Leaves are crucial as the primary sites for photosynthesis and for the exchange of materials and energy with the environment [61]. The palisade tissue, situated directly beneath the epidermis, absorbs most of the photosynthetically active radiation (PAR) and is more essential for photosynthetic productivity than the spongy tissue. The spongy parenchyma, characterised by multiple rows, primarily facilitates efficient gas exchange and transpiration. Additionally, it provides protection against temperature stress and passive moisture loss [62,63]. Therefore, the observed sensitivity of *J. regia* leaf tissues to cold stress was likely to lead to significant changes in photosynthesis parameters [64]. Furthermore, under light exposure conditions, photosystem II (PSII) is more susceptible to cold stress than photosystem I (PSI) [65].

The literature shows that a decrease in the photochemical quenching parameter known as Fv/Fm, which determines the potential maximum quantum efficiency of PSII, indicates photo-damage [66]. This was also observed in our experiments with *J. regia* microclones along with an ETR increase. An increase in PSII-mediated ETR was associated with low levels of stress [67], although at critical stress levels, a decline in this parameter was observed [68]. The observed adverse effects on the Fv/Fm index in our experiments could be indicative of the initial stages of degradation in the oxygen-releasing complex [69,70,71]. If the photochemical processes and electron transport in the chloroplast electron transport chain (ETC) are mismatched with electron collection rates, the excess electrons must be dissipated to avoid damage to the cell and the organism [72]. The literature’s evidence indicates that abiotic stress led to changes not only in energy absorption and ETR but also in energy dissipation, resulting in decreased PSII efficiency [73,74,75]. Consequently, less energy was used for photochemistry and more energy was lost as heat through mechanisms like xanthophylls (nonphotochemical quenching) [76].

In the current study, the quantum yield of nonphotochemical energy conversion in PSII, due to a downregulated light-harvesting function (regulated energy dissipation) Y(NPQ), significantly decreased under cold stress. In contrast, the quantum yield of nonphotochemical energy conversion in PSII that did not involve the downregulation of the light-harvesting function (unregulated energy dissipation) increased when microclones were exposed to cold stress. This provides a reliable indicator of the stress response in photosynthesis [77,78,79].

Temperature is a crucial physical factor that impacts secondary metabolites and supramolecular complexes through standard thermodynamic effects. Conceptually, any biologically active molecule could function as a thermos-sensor [80]. Studies from the 1980s and 1990s have established a correlation between cold damage and oxidative stress in plants. Low-temperature stress leads to cell membrane damage characterised by increased viscosity, gel phase formation, phospholipid breakdown, the accumulation of free fatty acids, and changes in protein and metabolite concentrations [81,82,83].

Reactive oxygen species (ROS), formed due to failures in the ETC, can trigger non-enzymatic reactions, further increasing their numbers. These reactions can subsequently alter the levels of secondary metabolites, such as polysaccharides, flavonoids, organic acids, alkaloids, and tannins, which exhibit significant anti-inflammatory, immunoregulatory, antibacterial, and antiviral activities [84,85,86]. In the current study, qualitative and quantitative analyses of the biologically active complexes of *J. regia* have provided evidence of these changes.

Recent studies have begun to actively explore the multicomponent nature of the plant antioxidant system, where all components interact functionally [64]. Modern theories suggest that low-temperature damage in plants starts with a rapid increase in cell membrane stiffness due to lipid bilayer phase transitions [85]. These changes in membrane structure and function lead to incremental alterations in chlorophyll-bearing leaf tissues, as observed in this study.

Phospholipids and glycolipids, the primary components of plant membranes, play a pivotal role in metabolism and enhancing plant resilience, especially under low temperatures [87,88]. These lipids, which have a glycerol backbone and two fatty acid “tails”, significantly influence membrane characteristics and the photoinhibition of PSII during stress [20,89,90]. Indeed, in the current study, the concentration of fatty acid esters significantly increased in *J. regia* microclones exposed to cold stress.

Terpenoids play a crucial role in lipid metabolism and accumulation [91]. In this study, exposure to cold stress led to an increase in the concentration of 3,7,11,15-tetramethyl-2-hexadecen-1-ol, commonly known as phytol. This increase in terpenoids, along with other volatile components like aldehydes, alcohols, and esters in green leaves, suggests their potential role in transmitting stress signals between plants [92]. Terpenoids can mitigate the effects of oxidative stress either through direct interactions with oxidants or by modulating ROS signalling [93,94,95]. Additionally, they may enhance the hydrophobic interactions between membrane proteins and lipids [95], protecting against destruction. Phytol, a component of chlorophyll, has a notable relationship with plant stress responses, especially under conditions that disrupt photosynthetic activity [57]. The observed increase in phytol content in *J. regia* microclones during cold stress underscores the significance of this finding.

Phenolic compounds are the predominant non-enzymatic antioxidants in plants, present in all tissues and cells [96]. The high concentration of these phenolics in *J. regia* leaves enhances their antioxidant properties. Phenolic compounds are predominantly synthesised in cellular locations such as plastids (chloroplasts and thioplasts), the endoplasmic reticulum, and the Golgi apparatus [97,98], highlighting the cellular infrastructure supporting their production. 

The leaf epidermis assumes a crucial role in adapting to adverse environmental conditions. Contemporary theories propose that only 5–10% of UV light penetrates beyond the epidermal layer, with the majority being absorbed by phenolic compounds in the vacuoles of epidermal cells. The observed reduction in the thickness of both the abaxial and adaxial epidermises during cold stress may result in an increased concentration of phenolic compounds, which typically elevate under stress conditions [20,56,99].

The antioxidant activity of phenolic compounds is largely determined via the number of hydroxyl groups on the aromatic ring [100,101,102]. Currently, about 10,000 phenolic compounds are known, which participate in various physiological processes and exhibit antioxidant properties [97,98]. However, the synthesis of these compounds during the early phases of plant ontogenesis, particularly in in vitro cultures under adverse environmental conditions, is not well understood [103,104].

Study findings show that cold stress significantly stimulates the production of stilbene, specifically (2,3-Diphenylcyclopropyl)methyl phenyl sulfoxide, in *J. regia* microclones. Stilbenes, a small subclass of phenolic compounds, are synthesised as a response to both abiotic and biotic stress factors. Stilbenes and their closely related secondary metabolites, flavonoids, may possess antioxidant properties due to their ability to scavenge free radicals and chelate metal ions involved in radical reactions [105,106]. Research indicates that stilbene biosynthesis is regulated via plant stress hormone signalling, ROS generation, calcium signalling, and the MAP kinase cascade [107,108,109].

Stilbenes, particularly (2,3-Diphenylcyclopropyl)methyl phenyl sulfoxide, exhibit significant biologically active properties, including cardioprotective, antibacterial, and antitumor effects [110,111,112]. Understanding the natural processes that control stilbene biosynthesis can aid in developing new plant protection strategies and in the commercial production of stilbenes [113]. However, the mechanisms governing stilbene biosynthesis have been relatively under-researched, making it crucial to explore the associated enzymes, genes, and the influence of stress factors on stilbene production [114,115,116]. Therefore, our findings that cold stress stimulates stilbene production in vitro in *J. regia* microclones hold considerable scientific and practical importance.

Pigments like carotenoids, melanin, and quinoid compounds are unique secondary metabolites with diverse ecological functions. While the protective roles of carotenoids and melanin against abiotic factors such as ultraviolet radiation, moisture deficiency, and other environmental stresses have been well documented [117], the ecological functions of quinone pigments are not as thoroughly explored. There is, however, limited evidence suggesting that the synthesis of various auto-oxidable naphthoquinone compounds serves as a protective response to stress through the shikimate pathway [118,119,120]. Additionally, it has been established that juglone biosynthesis can inhibit shoot growth, photosynthesis, respiration, and water transport in plants [120,121,122,123]. Moreover, the presence of naphthoquinones has been shown to stimulate the activity of antioxidant enzymes such as superoxide dismutase and catalase.

The findings from our study, which revealed a sevenfold increase in juglone concentration when *J. regia* microclones were exposed to low positive temperatures in vitro, are particularly significant. This substantial increase in juglone under stress conditions contributes greatly to our understanding of plant adaptation mechanisms to stressful environmental conditions. The data presented go beyond illustrating a biological system’s response to harsh external conditions; they also exemplify a model system that facilitates the development of effective technologies for the targeted synthesis of valuable secondary metabolites. This model can be used to create optimal conditions for the super-production of biologically active substances in vitro.

## 4. Materials and Methods

### 4.1. Plant Material and Growing Conditions

The study focuses on the Ideal variety (*Juglans regia* Ideal), which is specifically adapted for the Almaty region. This variety was imported from Uzbekistan and provided by a private farm in the Enbekshi-Kazakh district of the Almaty region, with certificate number SNJ326. The Ideal variety is characterised by its low-growing stature, reaching no more than 4–5 m in height. It bears large fruits, averaging 10 g in weight, with a light kernel that is easily removed due to its thin shell. Fruit ripening typically occurs between September and October, with a fruit yield from a single tree reaching up to 120 kg. Additionally, it exhibits frost resistance down to −30–35 °C and shows resilience to chlorosis.

For the establishment of aseptic cultures, explants underwent a sterilisation process involving a 5 min immersion in a soap solution followed by a 10 min treatment with 10% hydrogen peroxide. These procedures were conducted within a laminar flow hood, after which the isolated bud was placed onto a nutrient medium.

Adventitious and axillary buds from lignified shoots of *J. regia* were selected as the initial material for in vitro propagation. Following sterilisation, a bud was carefully isolated and placed on a modified DKW (Driver and Kuniyuki Walnut) [124] nutritional medium. This medium was adjusted to half the usual concentration of macro-elements and supplemented with the phytohormones BAP (1 mg L^−1^) and 1-naphthaleneacetic acid (NAA) (0.2 mg L^−1^), glucose (20 g L^−1^), and agar (7 g L^−1^), maintaining a pH of 5.7. The cultivation followed the standard protocol with a 16 h photoperiod (with illumination ranging between 6 and 9 LUX) and a temperature maintained at 24–26 °C. Transplantation to fresh nutrient media was performed every 20–30 days to ensure optimal growth conditions.

A genetically homogeneous planting material of *Juglandaceae* with an increased content of biologically active substances was obtained, and microsatellite SSR markers widely used for the identification of walnut plants were used in the work. The size of allelic loci WGA001, WGA005, WGA009, WGA069, and WGA202 varied between 178–192 bp, 240–252 bp, 237–252 bp, 160–184 bp, and 259–295 bp, respectively.

### 4.2. Analysis of Changes in the Elements of the Anatomical Structure

A root fixation was performed using 70% ethanol, and the preservative fluid used was Strasburger–Flemming’s mixture, consisting of 96% ethanol, glycerol, and water in a 1:1:1 ratio [125]. Anatomical samples were prepared with an MZP-01 microtome (Technom, Ekaterinburg, Russia), equipped with a freezing unit OL-ZSO 30 (Inmedprom, Yaroslavl, Russia). Microscopic images of the anatomical sections were captured using a Micro Opix MX 700 (T) microscope (West Medica, Wiener Neudorf, Austria) equipped with a CAM V1200C HD camera (West Medica, Wiener Neudorf, Austria). All anatomical data were collected using a 40× objective and involved 3–5 replicates, with each replicate consisting of three plants.

### 4.3. Analysis of Water Content Changes

To determine the moisture (water) content of plant materials, a 3 g sample was placed into a bottle that had been previously dried and tared. Obtained bottles were transferred to the drying oven set at the temperature of 100–105 °C and then heated for 30 min. After heating, the sample was cooled and weighed. Sample drying and cooling were continued, and weight was measured in 30 min intervals until a constant mass was achieved, indicated by a weight difference of no more than 0.1 g between two consecutive weightings. The moisture content of the raw material (*x*) was then calculated as a percentage using Formula (1):

(1) $$x=\frac{\left(m_{1}-m_{2}\right)\times 100}{\left(m_{1}-m_{0}\right)}$$

where *m*_0_ is the mass of an empty bottle (with a lid), dried to constant weight, measured in g and *m*_1_ and *m*_2_ are the masses of the bottle (with a lid) containing a sample before and after drying, respectively, dried to constant weight, measured g.

### 4.4. Photosynthetic Activity Determination

Photosynthetic activity was assessed by measuring fluorescence levels. Rapid Light Curves (RLCs) were recorded using a Junior-PAM fluorometer (Heinz Walz GmbH, Effeltrich, Germany) with actinic illumination at 450 nm. For each measurement, the fluorometer emitted eight saturation light pulses of 10,000 µmol m^−2^ s^−1^ at 20 sec intervals, while actinic light intensity gradually increased from 0 to 625 µmol m^−2^ s^−1^ [76]. The data for comparison were obtained from the last pulse of the light curve, where the readings across all considered parameters reached a plateau, ensuring the most objective difference in indicators.

The following parameters were calculated using Win-Control-3.29 software (Walz, Effeltrich, Germany): Fv/Fm—the maximum quantum yield of PSII photochemistry; PSII relative electron transport rate (ETR); Y(NO)—the quantum yield of nonphotochemical energy conversion in PSII caused by the downregulation of the light-harvesting function; Y(NPQ)—the quantum yield of nonphotochemical energy conversion in PSII due to the regulated dissipation of energy. In the study, measurements consistently targeted the middle third of the active leaf. All photosynthetic data were collected using a 40× objective with 3–5 replicates (one leaf each).

### 4.5. Sample Preparation for Phytochemical Analysis

For 30 days, 10 g of a plant sample was extracted with 50 mL of 96% ethanol. Each variant was prepared in at least three replicates.

#### 4.5.1. Qualitative Analysis

Various chemical tests were performed to detect biologically active compounds in extracts of *J. regia*, using standard methods with minor modifications [126,127].

Lead Acetate Test. Between 3 and 5 drops of a 10% solution of basic lead acetate were added to 1 mL of the extract, resulting in precipitation ranging from bright yellow to brown. This indicates the presence of compounds like phenols, phenolic acids, flavonoids, anthraquinones (yellow precipitate), hydrolysable tannins (brown precipitate), and flavones (brown-yellow precipitate).

Ferric Chloride Test. Between 1 and 2 drops of a freshly prepared 3% solution of iron (III) chloride were added to 1–2 drops of the extract. A green or blue-green precipitate indicates the presence of phenolic compounds. Green precipitate indicates a free 5-OH group and a two-row arrangement of hydroxy groups, and blue-violet precipitate indicates flavonoids, anthraquinones, phenols, phenolic acids, and tannins.

Oxalic Acid Test. Around 1–3 mL of a 10% solution of oxalic acid dissolved into a 1:1 mixture of acetone and water was added to 1 mL of extract. Bright colours indicate anthocyanins and anthocyanidins.

Ninhydrin Test. A total of 1 mL of extract was mixed with 0.5 mL of 1% ninhydrin solution followed by heating until a blue-violet stain appeared, which is specific to α-amino acids.

Aluminium Chloride Test. About 1–3 drops of a 1% aluminium chloride solution were added to 1 mL of the extract. An intensified yellow colour indicates the presence of flavonoids and other polyphenolic compounds with three ordinary OH groups, or OH...S(O)...OH-fragment and flavonol-3-glycosides.

Iron Ammonium Alum Test. Around 1–3 drops of a 1% solution of iron ammonium alum were added to 1 mL of the test solution. The resulting black-blue colour indicates the presence of condensed tannins, while a green colour indicates the presence of ortho-dioxy groups of any phenolic compounds.

The 1% KMnO_4_ Solution Test. Around 1–2 drops of a 1% solution of potassium permanganate were added to 1-3 mL of extract. The resulting purple precipitate indicates the formation of cocaine.

Gelatine Test. A 1% solution of gelatine was added to the extract, the resulting white precipitate indicates the presence of tannins. An excess of gelatine clears the turbidity.

Vanillin–Hydrochloric Acid Test (Zaprometov reaction). Around 1–3 drops of a 1% vanillin solution in concentrated HCl were added to 1 mL of the extract. The resulting yellow colour indicates the presence of flavones, while a range of pink colours indicates the presence of resorcinol and phloroglucinol derivatives.

#### 4.5.2. Quantitative Analysis

Tannins. The content of tannins was determined using a titrimetric method, with 0.2 M of KMnO_4_ as the titrant and indigo carmine sulphate as the indicator [128].

Flavonoids. The quantitative content of flavonoids was expressed in terms of quercetin, measured using a LEKI SS2107UV spectrophotometer (MEDIORA OY, Helsinki, Finland) [127].

Free Organic Acids. The content of free organic acids was determined with the titrimetric method using 0.1 M of NaOH as the titrant and phenolphthalein as the indicator. The results were calculated in terms of malic acid [128].

Alkaloids. The quantitative content of alkaloids was measured using a LEKI SS2107UV spectrophotometer (MEDIORA OY, Helsinki, Finland). Measurements were taken in a 10 mm cuvette at a wavelength of 420 nm, using a 2% sulfuric acid solution as a reference [129].

Polysaccharides. To determine the content of polysaccharides, an analytical sample of raw materials was extracted twice with water in a water bath with reflux. About 25 mL of the filtrate was mixed with 75 mL of 95% ethyl alcohol, heated in a water bath at 60 °C for 5 min, and centrifuged at 5000 rpm for 30 min in an OPN-8UHL4.2 centrifuge (AnalitPromPribor, Moscow, Russia). The precipitate was filtered, washed with 15 mL of 95% ethyl alcohol, and dried at 100–105 °C to a constant weight. The content of polysaccharides was then determined based on the weight of dry raw materials in % [130].

### 4.6. Gas Chromatography–Mass Spectrometry

GC–MS analysis of *J. regia* extracts was performed using an Agilent 7890A/5975C system (Santa Clara, CA, USA). The sample volume injected was 0.7 µL with an injection temperature of 280 °C, and the analysis was conducted without flow division. Chromatographic separation occurred on a DB-17MS capillary column, which was 30 m in length, with an inner diameter of 0.25 mm and a film thickness of 0.25 µm. The carrier gas (helium) was maintained at a constant flow rate of 1 mL min^−1^. The temperature programming for the chromatography started at 40 °C (held for 0 min) and ramped up to 280 °C at a rate of 5 °C min^−1^, with a final hold at 280 °C for 10 min. The total analysis time was 58 min. 

Detection was performed in the SCAN mode with an *m*/*z* range of 34–750. The Agilent MSD ChemStation software (version 1701EA, Santa Clara, CA, USA) controlled the GC system and facilitated the registration and processing of results and data. Data processing included determining retention times, analysing peak areas, and processing spectral information obtained from the MS detector. The identification of compounds was aided via the Wiley 7th edition and NIST’02 libraries, which contain over 550,000 spectra.

### 4.7. Statistical Analysis

The data analysis was conducted using RStudio software (version 2023.06.0 Build 421, RStudio PBC, Boston, MA, USA, 2023). Tukey HSD tests were performed for pairwise comparisons of the means, while an ANOVA was used to confirm statistical significance. Subsequently, the treatments were categorised by letter in descending order, and graphs were generated. Significance was declared at *p* < 0.05.

## 5. Conclusions

Pioneering comprehensive in vitro research on *Juglans regia* L. microclones was conducted to explore the effects of low positive temperatures on their anatomical, physiological, and phytochemical characteristics. These studies have identified significant changes in the synthesis of secondary antioxidant metabolites. GC–MS revealed that stress conditions substantially alter the metabolome of *J. regia* microclones. While the spectrum of metabolites was considerably reduced, there was a notable increase in the production of beneficial secondary antioxidant metabolites, including a sevenfold increase in juglone concentration. These findings are crucial for advancing walnut metabolomics and enhancing our understanding of plant responses to abiotic stressors, as well as elucidating the roles of individual metabolites in these processes.

## Figures and Tables

**Table 1:** Anatomy alterations in *J. regia* microclones.

	Parameter	Unit	Ctrl	Cold Stress	% to Ctrl	*p*-Value
	Stem anatomy					
Thickness	Epidermis	mm	0.031 ± 0.003	0.029 ± 0.003	−	**0.304**
	Collenchyma		0.064 ± 0.011	0.059 ± 0.007	−	**0.446**
	Sclerenchyma		0.150 ± 0.020	0.134 ± 0.004	−	**0.158**
	Parenchyma		0.262 ± 0.017 **b**	0.442 ± 0.103 **a**	169	<0.01
	Endoderm		0.038 ± 0.003 **a**	0.029 ± 0.003 **b**	76.3	<0.01
S	Conductive bundles	mm^2^	0.139 ± 0.010 **b**	0.249 ± 0.048 **a**	179	<0.05
	Phloem		0.086 ± 0.005 **b**	0.158 ± 0.009 **a**	184	<0.001
	Xylem		0.043 ± 0.005 **b**	0.076 ± 0.017 **a**	177	<0.05
d	Cell	mm	0.224 ± 0.010 **a**	0.197 ± 0.009 **b**	87.9	<0.01
Leaf anatomy						
Thickness	Adaxial epidermis	mm	0.039 ± 0.003	0.037 ± 0.002	−	**0.130**
	Abaxial epidermis		0.043 ± 0.004 **a**	0.035 ± 0.001 **b**	81.4	<0.001
	General mesophyll		0.185 ± 0.020	0.186 ± 0.017	−	**0.964**
	Palisade mesophyll		0.077 ± 0.006	0.076 ± 0.007	−	**0.970**
	Spongy mesophyll		0.087 ± 0.014	0.095 ± 0.009	−	**0.313**
S	Central vein	mm^2^	0.176 ± 0.016	0.170 ± 0.009	−	**0.409**
d	Midrib	mm	0.931 ± 0.064	0.869 ± 0.037	93.3	0.050 *
	Cell		0.088 ± 0.006 **a**	0.070 ± 0.004 **b**	79.5	<0.001

Notes: S—area; d—diameter; *—tendency at *p* < 0.1.

**Table 2:** Comparative qualitative analysis of biologically active substances in *J. regia* microclones exposed to low positive temperatures.

Reagent	*J. regia* Microclones	
	Ctrl	Cold Stress
10% PbAc_2_ (anthraquinones and others with ortho-hydroxy groups, hydrolysable tannins, flavones)	+	++
3% FeCl_3_ (phenolic compounds, tannins)	++	+++
10% oxalic acid (anthocyanins, anthocyanidins)	+	+
1% ninhydrin (amino acids)	++	++
5% NaOH (phenols, reduced forms of anthraquinones, 1,8-dihydroxy derivatives)	+	+++
1% aqueous solution of iron-ammonium alum (condensed tannins, ortho-dioxy groups of any phenolic compounds)	++	+++
1% AlCl_3_ (flavonoids, polyphenolic compounds with 3 ordinary OH groups, flavones, and flavonol-3-glycosides)	+	++
1% KMnO_4_	+	++
1% gelatine (tannins)	++	+++
1% vanillin in HCl (conc.) (Zaprometov reaction) (flavones)	+	++

Notes: ‘+++’—high content; ‘++’—moderate content; ‘+’—low content.

**Table 3:** Results of chromatographic analysis of *J. regia* microclones.

Retention Time, min		Substance	Class	Identification Probability, %		Content, %	
Ctrl	Cold Stress			Ctrl	Cold Stress	Ctrl	Cold Stress
16.25	−	4H-Pyran-4-one, 2,3-dihydro-3,5-dihydroxy-6-methyl-	Heterocyclic ketones	67	−	1.46	−
16.39	−	Benzoic acid, 2-formyl-	Aromatic CAs	70	−	1.25	−
17.67	−	1,2-Benzenediol	Polyhydric phenols	87	−	6.40	−
18.25	−	Benzofuran, 2,3-dihydro-	Monoaldehydes	71	−	1.06	−
20.54	−	2-Furancarboxaldehyde, 5-(hydroxymethyl)-	Heterocyclic alcohols	70	−	2.41	−
21.12	−	Hydroquinone	Polyhydric phenols	90	−	9.38	−
22.78	−	N-Acetyl-l-alanine ethylamide	Keto acids (oxo acids)	69	−	1.77	−
23.25	−	Phenol, 2,6-dimethoxy-	Phenol ethers	76	−	1.54	−
23.47	−	Ethyl 2,3-epoxybutyrate	Hydroxy acids	72	−	2.09	−
26.35	−	2-Pyrrolidinecarboxylic acid-5-oxo-, ethyl ester	Amino acid esters	87	−	3.65	−
28.05	28.03	1,4-Naphthalenedione, 5-hydroxy-	Naphthoquinones	82	83	7.50	49.92
28.17	−	D-Allose	Carbohydrates	88	−	3.52	−
−	28.59	Egenine	Isoquinoline alkaloid derivatives		70		1.68
29.09	29.07	3,7,11,15-Tetramethyl-2-hexadecen-1-ol	Phytols, terpenes	82	77	0.46	2.07
30.03	−	Ethyl α-d-glucopyranoside	Carbohydrates	84	−	8.29	−
31.17	−	3-Deoxy-d-mannoic lactone	Lactones	74	−	6.98	−
31.39	−	3-O-Methyl-d-glucose	Carbohydrates	70	−	3.16	−
31.54	−	1,2,3,5-Cyclohexanetetrol, (1α,2β,3α,5β)-	Cyclic PAs	68	−	15.25	−
32.49	−	Hexadecanoic acid	Saturated FAs	77	−	1.31	−
32.59	32.58	Hexadecanoic acid, ethyl ester	Saturated FAs esters	84	85	2.07	5.90
33.96	−	2,7-Anhydro-l-galacto-heptulofuranose	Heterocyclic compounds	80	−	3.53	−
34.49	−	d-Gluco-heptulosan	Heterocyclic alcohols	78	−	3.60	−
−	35.70	Dibutyl phthalate	Dicarboxylic acids esters	−	86	−	1.90
−	36.16	(E)-9-Octadecenoic acid ethyl ester	Unsaturated FAs esters	−	82	−	13.71
36.17	−	Ethyl Oleate	Unsaturated FAs esters	85	−	4.92	−
36.48	36.47	9,12-Octadecadienoic acid, ethyl ester	Unsaturated FAs esters	86	83	3.40	11.14
36.54	−	9,12-Octadecadienoic acid (Z,Z)-	Unsaturated FAs	77	−	1.29	−
−	36.96	9,12,15-Octadecatrienoic acid	Unsaturated FAs	−	70	−	1.71
−	38.03	4,4′-(Hexafluoroisopropylidene)diphenol	Diphenylmethanes	−	68	−	1.22
39.45	−	Hexadecanoic acid, 2-hydroxy-1-(hydroxymethyl)ethyl ester	PAs and saturated FAs esters	65	−	0.79	−
41.92	−	Oleic acid, 3-hydroxypropyl ester	Polyunsaturated FAs esters	74	−	1.18	−
42.97	−	Butyl 9,12-octadecadienoate	Unsaturated monobasic CAs	75	−	1.73	−
−	44.86	(2,3-Diphenylcyclopropyl)methyl phenyl sulfoxide	Stilbenes	−	69	−	10.74

Notes: FAs—fatty acids; PAs—polyhydric alcohols; CAs—carboxylic acids.

## Data Availability

The data are available upon request from the corresponding author.
